# Remote fruit fly detection using computer vision and machine learning-based electronic trap

**DOI:** 10.3389/fpls.2023.1241576

**Published:** 2023-10-10

**Authors:** Miguel Molina-Rotger, Alejandro Morán, Miguel Angel Miranda, Bartomeu Alorda-Ladaria

**Affiliations:** ^1^ Industrial Engineering and Construction Department, University of the Balearic Islands, Palma, Spain; ^2^ Biology Department, University of the Balearic Islands, Palma, Spain; ^3^ Institute for Environmental Agro-Environmental Research and Water Economics, University of the Balearic Islands, Palma, Spain; ^4^ Health Science and Technology Cross-cutting Department, Balearic Islands Health Research Institute (IdISBa), Palma, Spain

**Keywords:** precision agriculture, olive fruit fly pest, machine learning, support vector machine, random forest, computer vision, edge computing, remote sensing

## Abstract

**Introduction:**

Intelligent monitoring systems must be put in place to practice precision agriculture. In this context, computer vision and artificial intelligence techniques can be applied to monitor and prevent pests, such as that of the olive fly. These techniques are a tool to discover patterns and abnormalities in the data, which helps the early detection of pests and the prompt administration of corrective measures. However, there are significant challenges due to the lack of data to apply state of the art Deep Learning techniques.

**Methods:**

This article examines the detection and classification of the olive fly using the Random Forest and Support Vector Machine algorithms, as well as their application in an electronic trap version based on a Raspberry Pi B+ board.

**Results:**

The combination of the two methods is suggested to increase the accuracy of the classification results while working with a small training data set. Combining both techniques for olive fly detection yields an accuracy of 89.1%, which increases to 94.5% for SVM and 91.9% for RF when comparing all fly species to other insects.

**Discussion:**

This research results reports a successful implementation of ML in an electronic trap system for olive fly detection, providing valuable insights and benefits. The opportunities of using small IoT devices for image classification opens new possibilities, emphasizing the significance of ML in optimizing resource usage and enhancing privacy protection. As the system grows by increasing the number of electronic traps, more data will be available. Therefore, it holds the potential to further enhance accuracy by learning from multiple trap systems, making it a promising tool for effective and sustainable fly population management.

## 1 Introduction

Precision Agriculture for pest management requires constant monitoring of the target pest population as well as continuous evaluation of environmental conditions like temperature and humidity. Bactrocera oleae (Gmelin), known as the olive fruit fly, is a serious pest in the olive industry. If environmental conditions favour the proliferation of this tephritidae, losses from this pest might exceed 100% of productivity in a year. As a result, developing a system capable of collecting field data is critical for precise pest management.

The traditional monitoring system is based on flytraps. Those traps kill specific species of fruit flies, which are then manually collected and identified. The number of flies trapped are checked manually usually every week during the fruit fly season and then fortnightly during the winter months. The number of hours spent in this check task is huge and due to the manually data collection frequency, the time to detect an infestation is too large for flash responses. Therefore, developing a monitoring station to automate this manual trap checking will produce many benefits Martins et al. (2019). In addition, several environmental and public health problems appear when insecticides and off-target sprays are used extensively without adequate management. Weather parameters like air temperature and humidity levels in the spraying area are critical to determine the moment to spray and the duration of this process. The adult fly population is the insecticide target, and the weather conditions are important to decrease or increase the spray process effectiveness. In this sense, automatically monitoring those parameters in real time using computer-based platforms is important to adjust the spray activity.

In general, agricultural scenarios seem to be one of the most promising application areas for wireless monitoring station deployments due to the necessity of improving the agro-food production chain in terms of precision and quality. This involves a careful system design, since a rural scenario consists of an extensive area devoid of an electrical power supply and available wired connections. Automatic monitoring stations technology is introduced in Precision Agriculture strategy (PA) to obtain accurate real time field information and make accurate and optimum decisions Bjerge et al. (2023); Fasih et al. (2023).

Plant pest control remains one of the main research objectives of modern agriculture Shah and Wu (2019). The widespread use of insecticides at the field level is still the most common practice for the control of plant pests in general and for the fruit flies in particular Dias et al. (2018). However, its use is being restricted by official authorities due to its impact on the environment, human health, and the development of resistance in target pests. The use of PA for pest control has been applied to improve the control and/or detection of several pests, as examples: particularly sensitive maps are used to drive variable insecticide application for the control of certain insect pests Reay-Jones et al. (2019); hyperspectral imaging is used to detect fruit fly infestation in fruits Ding et al. (2021); or GIS technologies are used to implement user support systems to take more precise decisions about treatments of insect pests in the Mediterranean areas Goldshtein et al. (2021). In all these cases, a continuum of more accurate monitoring data produces a more accurate assessment of pest presence which, together with geolocation information, improves understanding of the spatial and temporal distribution of pest effects. In fact, the fast access to the information about pests is mandatory to accurately manage pests and diseases in agriculture Grasswitz (2019).

Since the monitoring of fruit flies is dependent on fly identification, the first fruit fly identification platform was proposed by Pontikakos et al. (2012) as a combination of traditional manual inspection process and the computer-based platform for storing the trap checking results. The proposed computer-based platform can perform olive fruit fly evolution analysis and treatment prediction considering weather conditions. Although the manual trap inspection is also required, the automatic analysis of data combined with weather conditions allows determining the best period to apply the spray treatment and the areas to be considered in the treatment.

The second one is related to solve the identification process and reduce the time needed to check the fly traps. The authors in Bjerge et al. (2023); Fasih et al. (2023) describe a procedure to identify the fruit fly using image segmentation techniques using a camera as a sensor and some computing process to obtain the identification results. Although, the procedure is proposed using a MacPhil trap. In a MacPhil trap, the fly can be over or in the liquid introducing some additional difficulty for accurate fly identification process in comparison with using sticky traps. The sticky trap retains the flies on the surface of the trap plane and increases the possibilities to take an adequate photograph for identification purposes.

This is where computer vision and Artificial Intelligence (AI) come in. It can analyze the photo and identify the olive fly, reducing the time it takes to check the traps and automating the process. As a result, the farmer’s workload is reduced. Advances in image identification techniques have paved the way for the use of AI in this field. Although Deep Learning (DL) is the most commonly used technique, Krizhevsky et al. (2017), and there are examples of their effectiveness, Victoriano et al. (2023); Uzun (2023), this article discusses classical machine learning (ML) approaches. This is because DL requires a large dataset to achieve good results, and such a dataset is currently unavailable. It is also computationally expensive. Therefore, the study will focus on the ML algorithms Random Forests (RF) and Support Vector Machines (SVM).

This work shows the design and implementation of a real time automated low-cost olive fruit fly smart trap, will now be referred to as e-trap throughout this article. The main novelty is the use of ML for image identification, in addition to the connection through a GPRS link with a cloud-based platform described in Miranda et al. (2019). In particular, it is explored how RF and SVM can improve efforts to reduce the use of pesticides against the olive fly to prevent crop loss and monitor it remotely.

## 2 Materials and methods

The smart trap approach consists of a photographic camera for image capture, a linux-based electronic system to implement the algorithms to recognize olive fly adults, a solar-based power system, and an ambient relative humidity/temperature sensor. The sensor and picture data collected by the smart trap is processed and stored allowing *in-situ* access in case of communication lost.

**Figure 1:**
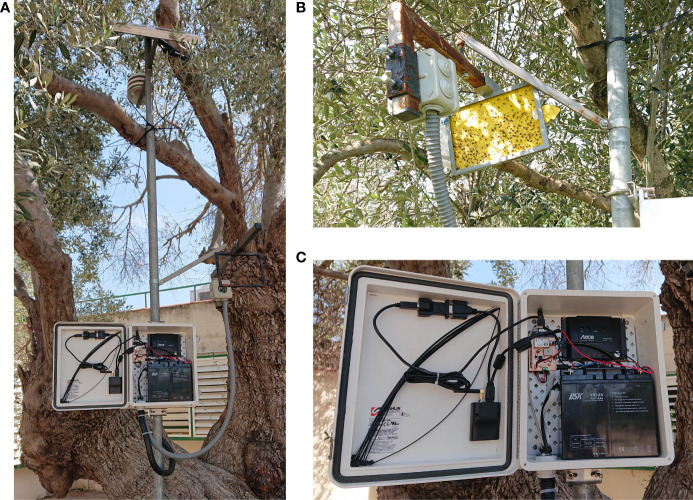
Electronic components of the e-trap. **(A)** E-trap with solar panel, Stevenson screen to protect the temperature and relative humidity sensor, battery and electronics. **(B)** Camera placed in front of a Rimi^®^ trap. **(C)** Battery and electronics.

The solar panel and the Stevenson screen for the humidity and temperature sensors are at the upper part of a metal pole see **Figure 1A**. The battery, transmitter system, and controller are included in a box just in the middle part, as **Figure 1A** shows. The controller system and the transmitter module are in the middle box for weather condition protection. In addition, **Figure 1B** shows the sticky trap supported by a metal pole, including a junction box with a camera installed in front of the trap. This camera is connected to the controller system for image capture and power supply. Finally, the lower part of the metal pole will be used to nail the pole on Earth and thus have a first fastening point to finish tying the pole to the strongest olive branches. In this way, the metal pole will be stable and tied up during the measurement period without disturbing the agricultural machines and workers between olive trees.

### 2.1 Sensors and camera

The designed prototype includes a temperature, a relative humidity sensor, and a camera serial interface (CSI). The two sensors (model DHT22) installed in the upper part of the pole will be connected and powered from the controller box. This sensor has enough resolution in both parameters, see **Table 1**. The DHT22 device provides a new value each 2 seconds with reduced energy consumption ratio. The controller system is designed to measure and save in local storage memory the temperature and humidity values each minute. But, only the maximum, minimum and the average values are transmitted to the cloud server every hour including the exact timestamp. This methodology reduces the amount of data to be sent to the server and filters the unwanted values (aberrant values or errors in communication with the sensor), storing the information on the station for post-analysis and maintenance purposes.

**Table 1:** Specifications of sensor and camera elements.

Parameter	Value
Sensor voltage supply	3.3 Vdc ≤ Vcc ≤ 6 Vdc
Sensor output type	Digital
Temperature range	-40°C to 80°C
Temperature accuracy	± 0.5°C
Temperature resolution	0.1°C
Humidity range	0% to 100% RH
Humidity accuracy	2% RH
Humidity resolution	0.1% RH
Sensor measurement period	2 s
Camera resolution	2592 x 1944 pixels
Camera focus	Fixed focus
Camera dimensions	25 x 20 x 9 mm

The camera used is a CMOS sensor Omnivision 5647 with removed IR filter (see **Table 1** for camera specifications). It is connected and powered by the controller system using a CSI bus. The cable between camera and controller is 1.5 meters long, allowing to determine the most adequate position of sticky trap without restrictions of distances, see metal arm where sticky trap and camera are fixed in **Figure 1B**.

The camera is the most energy demanding device in the proposed e-trap system apart from the 4G modem. Therefore, it is powered on during the instant to take the photo, afterwards, it remains turned off. The instant when taking the photography can be adjusted considering the sun position and the amount of light available. The smart trap has been programmed by default, to take three photos when the sun is around the upper level, so the sunlight intensity will be the highest producing the highest image contrast. The three photos will be taken around midday hour with a delay of 30 minutes between each photo. In addition, users can change the timing of the photo at any time to capture the best quality photo depending on the locations and shadows on the sticky trap surface.

Photographies are taken only three times a day because this is not a real-time application. Here the goal is to infer and report the insect population without being on the field. In addition, since the system is not perfect, it is convenient to take several photographies, three in our case, to filter errors and increase the amount of training data.

### 2.2 Controller and communication system

The controller system is one of the most important parts of the smart trap. It manages sensor, camera, data transmission and performs the fly identification task. All these tasks require enough computer resources, low energy consumption and system flexibility. In this work a Raspberry Pi B+ is selected to supply the required hardware requirements in combination with the Raspbian OS lite version. The selected platform is flexible enough to manage all the tasks reducing the number of active processes and power consumption, while image processing software can be implemented using open-source resources like OpenCV, Bradski (2000).

The communication module consists of an Airlink GL8200 modem connected to the controller system using the serial port interface (SPI). The communication uses flux control to obtain maximum transmission velocity ratios (115200 bps). The modem module is compatible with standard AT commands and can allow server connections using standard internet protocols like File Transfer Protocol (SFTP), Hypertext Transfer Protocol (http) and Network Time Protocol (NTP) between others. The NTP protocol is used to maintain and update the local real time controller (RTC) enabling a time-based schedule of the tasks. The http protocol enables the connection with the remote server to store the sensor data and the fly count result on the remote database. In case of necessary, the SFTP protocol allows uploading images to the server for validation purposes with the penalty to increase the energy consumption available at the smart trap. In any case, a SD storage disk is used to save all sensor data, fly count and images. Therefore, the data will remain in the smart trap in case communication fails and can be accessed manually visiting the trap location during sticky trap maintenance.

### 2.3 E-trap firmware

The e-trap controller is designed using the Raspian Lite operating system implementing a time-scheduled management. The different e-trap tasks are executed using the Cron task manager embedded in the Unix systems. In this way, the e-trap is configured to work alone without expecting interaction from remote infrastructures.

**Figure 2:**
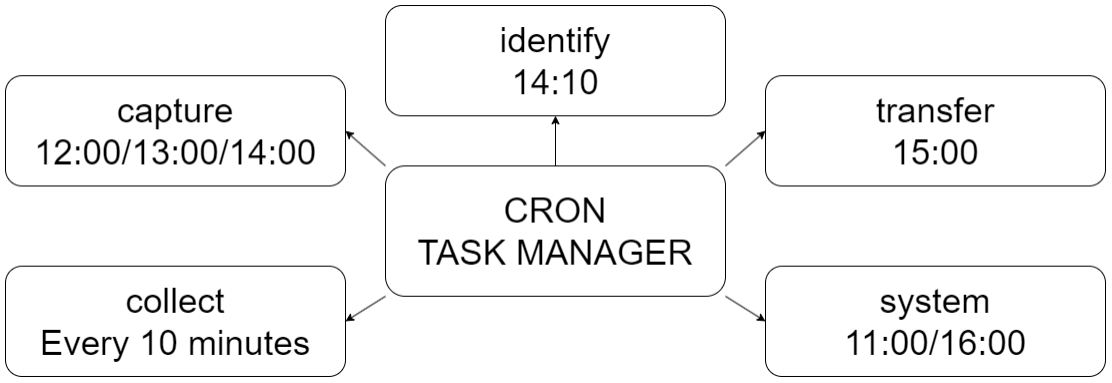
E-trap firmware flowchart showing the five main tasks and their execution times.

The e-trap firmware is divided into five main tasks as shown in the functional diagram in **Figure 2**. All tasks are lunched using the Cron manager, Kernighan and Pike (1984). The first task, called “system”, maintain the controller date and time updated, check the battery level, peripheral power supply management and rebooting-based strategy to avoid software issues. The second task, referred to as “collect”, is related to sensor access, and collects temperature and humidity values from DHT22 sensor storing it timestamped in a local file using CSV format. The third task, named “capture”, takes a picture adjusting the exposition time and white balance level to optimize the resolution and the quality of the picture. The fourth task, termed “identify”, analyzes the obtained images, and try to identify the number of flies trapped. This identification process is explained in the next section. And finally, the fifth task, called “transfer”, is responsible to establish LTE communications, to send the sensor data file to the remote server and to attend to the remote requirements (send the picture file or software update).

Each task of the e-trap software is launched by Cron daemon at different time during the day. Therefore, each task is implemented independently of the other tasks avoiding that one task stop the rest of tasks. In fact, meanwhile the Cron daemon is running, the tasks are initiated and terminated without interaction between them.

It is important to note that the “system” task is executed twice a day. The first time it reboots the controller to get a fresh system after one day of continuous operation. The second execution of the “system” task (@16:00) will shut down all peripherals not related to the collection task. With this procedure, the power consumption of Raspberry Pi platform is minimized until the next day’s reboot.

## 3 Data collection and generation

### 3.1 Dataset collection

**Figure 3:**
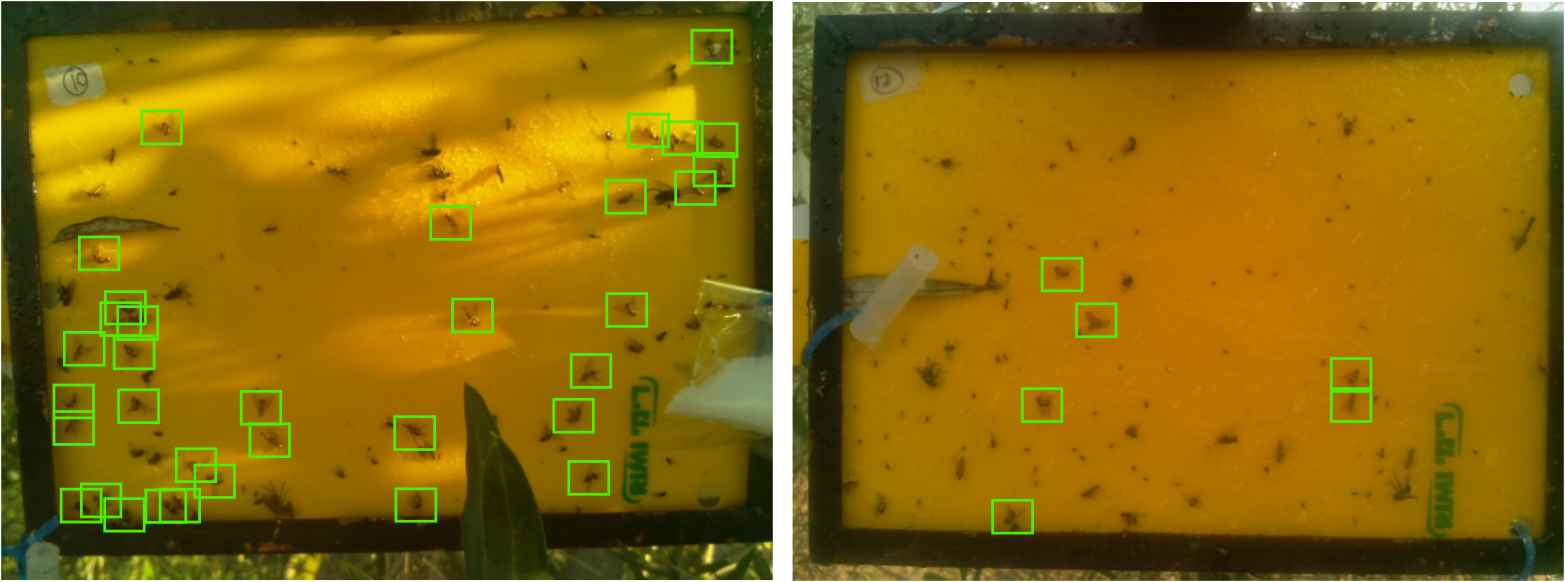
Example targets from N10 and N17 sticky traps.

This article uses images of the two e-traps identified as N10 and N17. These traps were placed in the olive fields of the “Institut de Recerca i Formació Agroalimentària i Pesquera de les Illes Balears” (IRFAP) in Mallorca, Spain. The OV5647 camera, which is already integrated in the e-trap itself, was used to capture the images. The resulting images have a resolution of 1600 pixels wide by 1200 pixels high, 3 RGB channels, 24-bit depth, and were saved in.jpg format. Note that the physical position of the traps in the olive trees was similar but not exactly the same, resulting in differences in the final image. The dataset consists of a total of 62 images, 45 generated by N10 and 15 generated by N17. **Figure 3** shows an example of an image taken by each of the traps and **Table 2** shows all this data summarized.

**Table 2:** Dataset collection parameters.

Parameter	Value
e-traps count	2 (N15 & N17)
Sticky trap images count	45/15 (N10/N17)
Location	IRFAP, Mallorca, Spain
Resolution	1600 × 1200 × 3
Depth	24-bit
Format	.jpg

**Figure 4:**
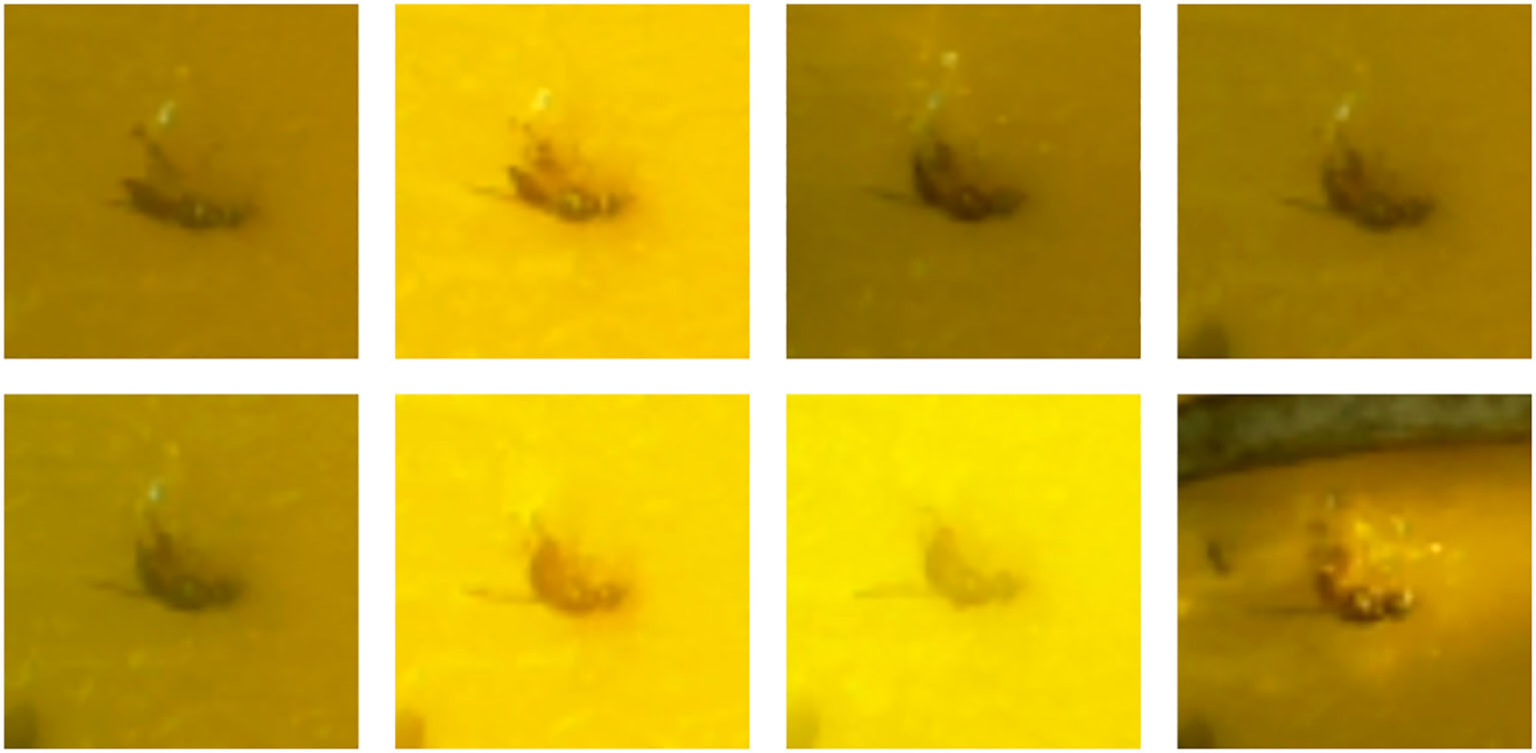
Example of the same olive fruit fly from 8 to 15 October on N17.

By taking a photo every day until the sticky pad is replaced, the observation reveals the emergence of new flies alongside the already trapped flies that persist over time. **Figure 4** shows how this allows us to know how the same olive fly is observed with different lighting, thus performing the data augmentation (DA) technique in an organic way and allowing the classifier model to learn which features have the highest priority in defining the fly for its correct classification. The application of this technique is common in the AI world, since it allows to face the problem of lack of data to train, and in the PA world it is no exception (Brilhador et al. (2019); Fawakherji et al. (2020)); (Shorten and Khoshgoftaar (2019)).

### 3.2 Dataset generation

The 45 images from N10 were used to train the classifier models. Classifier test was performed on the remaining 15 images from N17. This was advantageous because the classifier model never knew the training data and could even be given different e-trap positions and luminance conditions with respect to N10. In summary, it was possible to test whether a single e-trap could be used to generate a first scalable smart trap system capable of localizing and classifying the olive fruit fly.

After studying all the available images to train the classifier model, the dataset consisted of 501 olive flies, 368 flies of other species or very similar insects, and 611 different elements such as the bag or tube with the olive fly attractant, the brand of the adhesive panel, holes in the panel, other insects, shadows due to different lighting, trap identifier, etc., all of 32 × 32 × 3 pixels. All of these were grouped into two groups, “olive fly”/”others”, resulting in a data set with a ratio of 501 “olive fly” and 979 “others” samples. All these dataset values are summarized in **Table 3**.

**Table 3:** Training, validation, and test set sizes for the cropped images. Note that the training size refers to the already augmented data and the percentages refer to the sum of these augmented samples.

Parameter	Total value	Train value (90%)	Validation value (10%)	Test value
Olive flies	501	451	50	6
Other species flies	368	332	36	17
Other elements	611	550	61	14

A 9:1 ratio was used for training and validation of the models, i.e. 90% of the samples are used for training and 10% for validation. In addition, in order to have more working data, basic DA techniques that could be present in the nature of the project were applied: vertical image flipping, horizontal flipping, 90° rotation, and changes in the brightness and contrast of the images. These actions allowed us to enlarge each image up to 2^4^ = 16 new alternatives. In addition, it is worth highlighting these DA techniques based on basic image manipulations are considered “safe” for this application because the label is always preserved Shorten and Khoshgoftaar (2019).

Two conditions were set for this DA process: first, between zero and ten new images could be generated, this number being random for each sample. Second, each DA technique could occur with a 50% chance. In this way, the augmentation would not be homogeneous, thus preventing the model from learning repetitive patterns. This action eventually increased the training data set from 1332 to 8069 samples, and all AI models used it, so that the result comparisons for different models are not biased by the dataset.

Finally, the test images from N17 were simply labeled to match the image provided by the e-trap to simulate the real system process.

## 4 Machine learning classification models

As mentioned earlier, due to the size of the dataset, the final algorithms selected for this article were RF and SVM. These ML methods and their validation would be the focus of this section.

### 4.1 Random forest

Random Forest, introduced by Breiman (2001), is a supervised learning algorithm used for both classification and regression tasks. It is an ensemble method that combines multiple decision trees to make predictions. Each decision tree in the RF is built independently on a different subset of the training data, and the final prediction is made by aggregating the predictions of all the trees.

Here’s how RF works:

1. Data Preparation Given a collection of training examples denoted as ${\left[(x_{i},y_{i})\right]}_{i=1}^{n}$ , where *x_i_* represents the input features and *y_i_* represents the corresponding target labels, RF starts by randomly selecting subsets of the training data with replacement. These subsets are known as bootstrap samples.
2. Building Decision Trees: For each bootstrap sample, a decision tree is constructed independently. At each node of the decision tree, a feature subset is randomly selected, and the split that optimally separates the data based on some criterion (e.g., Gini impurity or entropy for classification, Jost (2006), mean squared error for regression, Langs et al. (2011)) is chosen. The tree continues to split the data until a stopping criterion is met, such as reaching a maximum depth or minimum number of samples required to split further.
3. Ensemble Prediction: Once all the decision trees are built, predictions are made by each tree on unseen data. For classification tasks, the class with the majority of votes among the trees is selected as the final prediction. For regression tasks, the average of the predicted values from all the trees is taken.

RF offers several advantages over individual decision trees:

- Ensemble Effect: By aggregating predictions from multiple decision trees, RF reduces the risk of overfitting and provides more robust predictions.
- Feature Randomness: Randomly selecting a subset of features at each node helps to decorrelate the trees and capture different aspects of the data.
- Out-of-Bag Evaluation: As the trees are built on bootstrap samples, the instances left out in each sample (out-of-bag instances) can be used for validation without the need for an additional holdout set.

In summary, RF is a versatile and powerful algorithm that combines the predictions of multiple decision trees to achieve high accuracy and robustness in both classification and regression tasks. It is particularly effective when dealing with complex data and can handle a large number of features.

### 4.2 Support vector machines

Support vector machines (SVM), introduced by Vapnik and Chervonenkis (2015), are also supervised learning models used for classification and regression analysis. The term SVM typically does not refer to a linear SVM, but rather to the use of kernel methods, Sánchez A (2003).

Given a collection of training examples denoted as ${\left[(x_{i},y_{i})\right]}_{i=1}^{n}$ , and a kernel function denoted as *K*, each *y_i_* belonging to the set [−1, +1] represents its categorization into one of two categories. An objective function of the SVM is used to solve the optimization problem defined as follows:


(1) $$\underset{\alpha }{\max}\left[\sum_{i=1}^{n}\alpha_{i}+\sum_{i,j=1}^{n}\alpha_{i}\alpha_{j}y_{i}y_{j}K(x_{i},x_{j})\right]$$

subject to the constraints:


$$0\leq \alpha_{i}\leq C$$

$$\sum_{i=1}^{n}\alpha_{i}y_{i}=0$$

Here, the Lagrange coefficients *α_i_* are involved, and the constant *C* is used to penalize training errors present in the samples.

An SVM training algorithm constructs a model that classifies new examples into one of two categories, acting as a non-probabilistic binary linear classifier. The SVM model represents the examples as points in a space in which they are mapped to ensure a clear gap that maximizes its width between the different categories. Then, new examples are projected into the same space and their categorization is predicted based on which side of the gap they fall. As mentioned in the introduction, the choice of the regularization parameters *α_i_* and the form of the kernel function $K\left(x_{i},x_{j}\right)$ have a significant impact on the performance of the SVM. These factors are thoroughly considered and extensively discussed in the comparative experiments.

### 4.3 Model validation

When building a model, there are several parameters to consider, and depending on how they are combined, the results may vary. In addition, there is a stochastic variable in the selection of data that may or may not favor the final result.

Therefore, the techniques used in this article can be grouped into two. (i) Grid search, to find the combination of hyperparameters that give the best results. (ii) Cross validation, to perform the process *k* times with different combinations of data, thus validating that the response of the classifier model is general and not specific to a single combination of data.

The metrics used for validation were: confusion matrix, accuracy, precision, recall, f1-score, Receiver Operating Characteristic (ROC) curve and the Area Under the ROC Curve (AUC).

#### 4.3.1 Confusion matrix

Measures the performance of a classification model by summarizing the number of true positive (TP), true negative (TN), false positive (FP), and false negative (FN) predictions in tabular form.

#### 4.3.2 Accuracy

This metric measures the proportion of correctly classified images out of the total number of images in the dataset.


Accuracy=((TP+TN)/TotalImages)∗10

#### 4.3.3 Precision

It measures the proportion of correctly predicted positive instances out of all instances predicted as positive.


Precision=TP/(TP+FP)

#### 4.3.4 Recall

The recall metric measures the ability of a model to correctly identify positive instances out of all the instances that are actually positive.


Recall=TP/(TP+FN)

#### 4.3.5 F1-Score

The F1 score is a metric that combines precision and recall to provide a single measure of a model’s performance in classification tasks, including image classification. It takes into account both the false positives and false negatives to assess the balance between precision and recall. The F1 score is calculated by


F1score=2∗(Precision∗Recall)/(Precision+Recall).

#### 4.3.6 ROC curve

The ROC curve is created by plotting the true positive rate (TPR) against the false positive rate (FPR) at various threshold settings. The TPR represents the recall or sensitivity (correctly predicted positive instances), while the FPR represents the proportion of negative instances incorrectly classified as positive.

#### 4.3.7 AUC

The AUC measures the performance of a model in terms of its ability to discriminate between positive and negative instances across different classification thresholds.

## 5 Image approach: fruit fly detection

Identifying the olive fruit fly in the e-trap images involved a number of challenges. The first was the lack of images available to train and validate the AI model. The second was the ability to distinguish the olive fruit fly from other fly families or dark elements that might appear in the images. Finally, the third was related to the processing power and energy consumption allocated for inference, in this case the target device was a Raspberry Pi B+.

The usual way to perform this process of object detection on an image is usually done by applying convolutional neural networks (CNNs). An example of this is the recent publication by Jia et al. (2023), where they apply the YOLOX-m network for the localization of different green fruits, such as green apple and green persimmon, among the leaves of trees, which can also be green. Other examples include the recognition and counting of bananas by Wu et al. (2021, 2023). The reason for applying this technique is mainly due to its ability to extract physical and temporal features from the images. However, in this paper, the CNNs path is discarded because the challenges mentioned in the previous section become clearly latent. State of the art CNNs require large datasets to train the model, which has not been available so far, and the computational process is expensive for some devices such as a Raspberry Pi B+ without external aids like a hardware accelerator.

The working dataset is considered small compared to the usual benchmarks for these tasks. For example, MNIST with 60,000 training images, CIFAR-10 and CIFAR-100 with 50,000 images each or Imagenet with 1.2 million training images (LeCun et al. (1998); Krizhevsky and Hinton (2009); Deng et al. (2009)).

Due to this challenge, in this article it was decided to finally apply classification methods based on traditional ML techniques. Although such models are mainly used for tabular data, present less overfitting when working with small amounts of data. In addition, since the model complexity is usually lower, in general, power consumption is lower too. **Table 4** shows the different models tested in a first step. It is observed that for the same set of training data and all the metrics of the ML models are clearly superior to those of the DL models. Therefore, it was decided to investigate the different ML models in more detail.

**Table 4:** Olive fly classification performance metrics for different traditional ML and DL approaches.

Model	Type	Accuracy	Precision	Recall	F1-score	AUC
Random Forest	ML	0.85	0.84	0.85	0.85	0.85
SVM	ML	0.81	0.80	0.80	0.80	0.80
Decision Tree	ML	0.75	0.78	0.75	0.75	0.77
VGG16	DL	0.59	0.68	0.69	0.39	0.54
MobileNet	DL	0.59	0.68	0.69	0.39	0.54
Xception	DL	0.58	0.68	0.69	0.38	0.53

The use of ML techniques for image processing is not new, Wang et al. (2021) concluded that traditional ML has a better solution effect on small sample data sets. Researchers such as Mekha and Teeyasuksaet (2021) have already studied the use of different ML algorithms for the detection of diseases in rice leaves, concluding that the application of RF was the one that gave them the best results. Another example are Liu et al. (2017), which proposes the use of the SVM algorithm for image classification in remote locations, as in our case, instead of using DL.

Performing fly detection with traditional ML methods was a new challenge. Some pre-trained DL models for object detection already have this built-in function, capable of locating and classifying objects, as well as understanding the overlap between different possible locations of the same object (Milioto et al. (2018); Prasetyo et al. (2020); Rong et al. (2022)). In our case, the solution was to first apply image processing that takes advantage of the contrast between the yellow background of the trap and the dark color of the fly to distinguish where the different elements to be classified appear. Finally, all that remained was the ML classification process for each of the elements found.

Since RF and SVM gave the top-2 better performance metrics compared to other models, it was decided to combine them to improve classification performance. Therefore, it is validated that the element is an olive fruit fly if both models assert that the element is an olive fruit fly.

**Figure 5:**
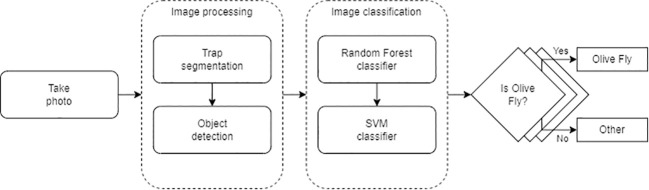
Inference pipeline.

**Figure 5** shows the logical flow. First, the image is captured. Second, the image is processed by segmenting the trap to avoid possible false positives and locating the elements that appear in the e-trap. Third, each element is classified one by one by applying RF and SVM. Finally, if both validate the classification, it is marked on the image.

## 6 Results

This section presents the results of the study. In the previous points, it was mentioned that CNNs are not able to provide accurate results due to the small training dataset. Therefore, classical ML solutions are compared with CNNs solutions.

### 6.1 Machine learning and deep leaning results

As mentioned above, the challenges of the project were: mainly how to deal with the limited training data available, and also whether it is possible to develop an accurate classifier model taking into account the low computational capacity of the Raspberry Pi B+. **Table 1** shows the metrics of the different models proposed in the first phase of the project.

As evident from the analysis, there are six evaluated models, comprising three classical ML algorithms and three CNN models. The ML algorithms are the already mentioned RF and SVM, and also the Decision Tree algorithm, which already includes the RF, as mentioned above.

On the other hand, the CNNs include the VGG16, Mobilenet, and Xception models (Simonyan and Zisserman (2014); Howard et al. (2017); Chollet (2017)). Models that are widely used for image classification due to their good results. For example, the work of Subramanian and Sankar (2022), where they compare this CNN model and others for coconut maturity detection. Or the work of Sehree and Khidhir (2022) that classifies olive trees from unmanned aerial vehicle images.

Looking at **Table 4**, the superiority of the ML becomes evident, maintaining an accuracy of no less than 75%, compared to the DL, which does not achieve more than 60% accuracy in any case due to the limited availability of data.

As mentioned in section 3.2 *Dataset Generation*, the validation data come from N10, so the metrics will always tend to be higher than the test metrics, which comes from e-traps unknown to the model. **Table 4** also shows the AUC value, DL models tend to be around 0.5, which could lead us to think that they are doing a random classification.

At this point, it was decided to take the two best results and test them as if the system was already in production.

### 6.2 Random forest and support vector machines analysis

**Figure 6:**
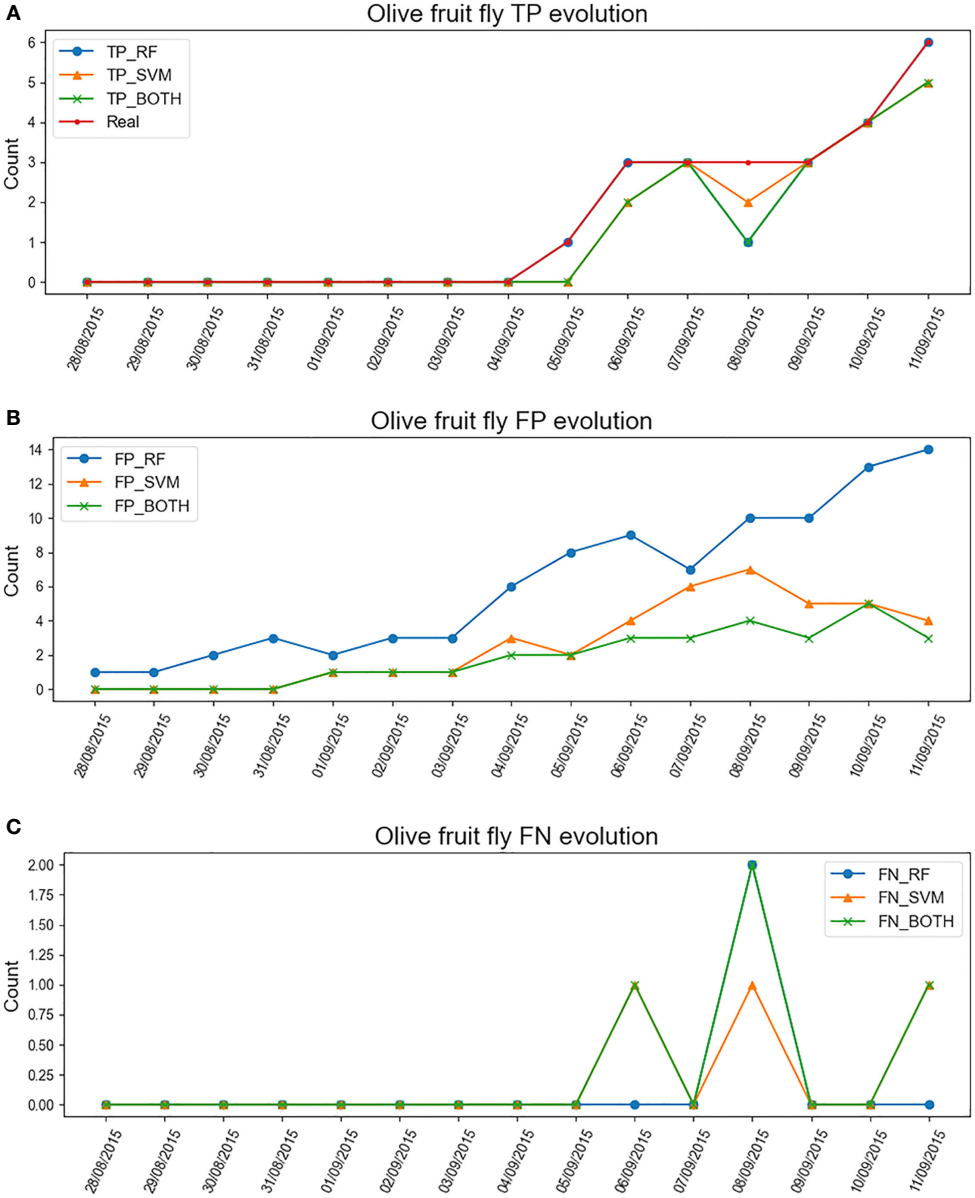
Evolution of the number of olive flies detected in the sticky trap as a function of time using different ML classifiers for the N17 e-trap. **(A)** TP evolution of RF classifier, SVM classifier and their combination together with the real count. **(B)** FP evolution of the same classifiers. **(C)** FN evolution of the same classifiers.

**Figure 6** shows the results of the two-week evolution of trap N17 from no flies to six flies. The **Figure 6A** refers to the true positives (TP), i.e. the correct classification of the olive fly by the different models. And the **Figures 6B, C** refer to the false classifications of the fly, the false positives (FP) refer to the elements that the model classified as flies and they are not, and the false negatives (FN) refer to the elements that are flies and the model discarded them. The final hyperparameters used in RF were: max depth of 20, min samples split equal to 5, and 3 estimators. And the final SVM hyperparameters were A polynomial kernel, C equal to 0.1, and gamma equal to 1.

#### 6.2.1 RF classifier

This model tends to classify most items that resemble an olive fruit fly as “Olive Fly”. After examining the images, one may conclude this is because the RF model is not able to differentiate whether a fly belongs to the olive fruit fly species or not, so its FP rate tends to rise and conversely the FNs are very low.

#### 6.2.2 SVM classifier

The graphs show how this model is more cautious about RF in determining whether an object is an olive fly or not. Therefore, its FP rate is lower, but it increases the FN discriminating flies that were correct.

#### 6.2.3 RF+SVM classifier

Finally, combining the two models allows for more accurate classification. The FNs go down even further, in exchange for the fact that if an item is claimed to be a olive fly, it is much more likely to be so.

## 7 Discussion

**Figure 7:**
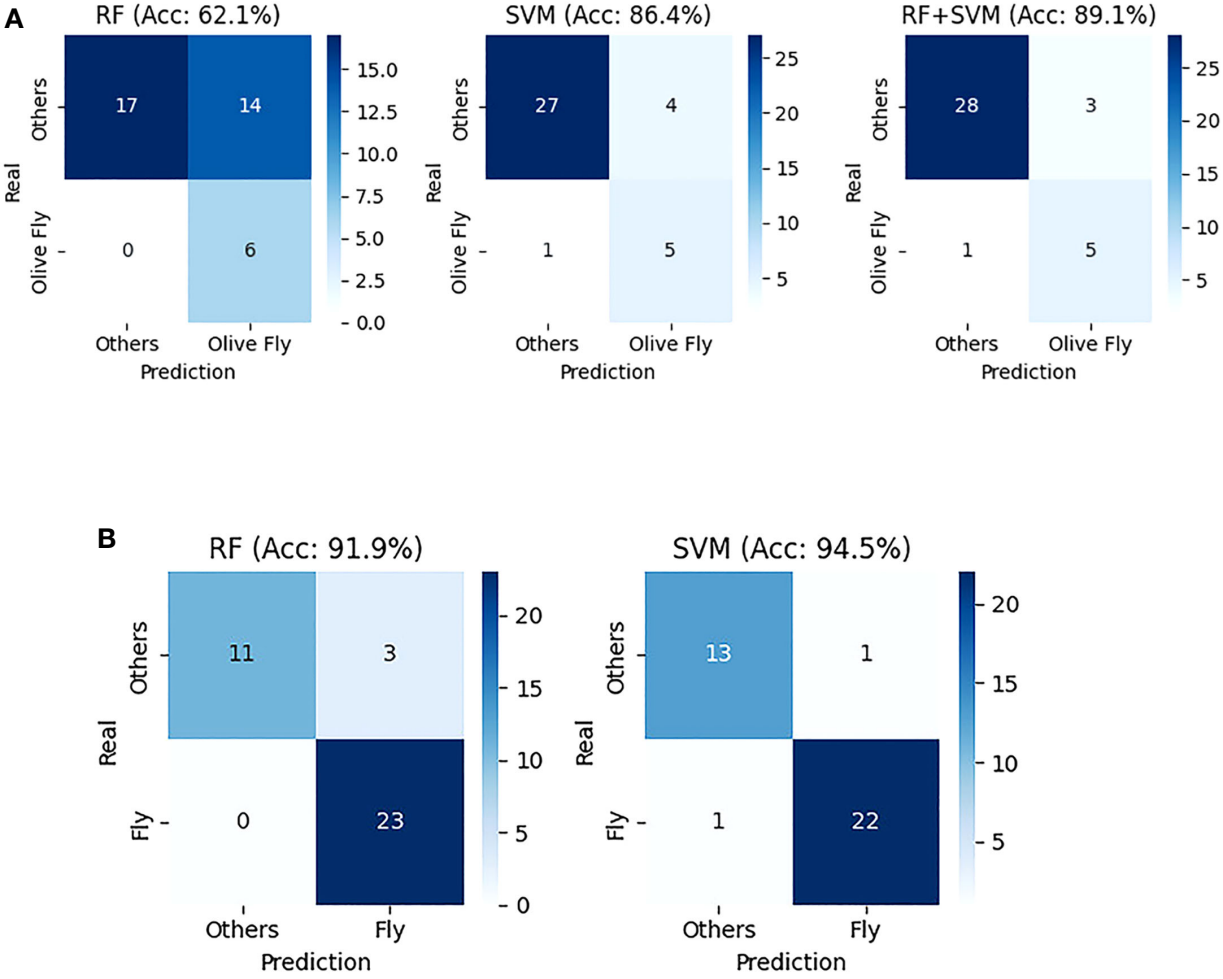
RF and SVM metrics on the N17 image from the 11th of October. **(A)** Confusion matrix comparison of RF, SVM, and “RF+SVM” models for olive fly classification. **(B)** Confusion matrix comparison of RF and SVM models for classification of all fly species.

In this study, an intelligent system capable of detecting the olive fly using non-invasive techniques was developed. Two models were created with an accuracy of 62.1% for RF and 86.4% for SVM, **Figure 7A**, using only the data of two traps, one for training and the other to validate the models.

While RF would be the first to warn of a possible fly infestation. SVM proved to be more conservative in stating whether or not there was a fly in the sticky trap. In addition, a third option was also presented too, the combination of both models to be able to combine the best of each and achieve a higher accuracy of 89.1%, as shown in **Figure 7A**.

It has been shown that it is also possible to control the olive fly using classical ML techniques. Allowing deploy this intelligent systems faster than if the detection were performed using CNN techniques. And consequently understand the status of the crops before and remotely observe the evolution of the fly population, **Figure 6A**. In addition, the robustness achieved using ML is reflected in **Figure 7B**. Here, the performance of both models is shown when trying to classify only flies, regardless of the species. As can be seen, the accuracy of both algorithms increases to 91.9% for RF and 94.5% for SVM.

Therefore, this project demonstrates the application of ML on an e-trap system that facilitates the control tasks to the experts, being able to reduce the number of times they should go to the fields to make the manual count of the flies, as well as providing additional information not to go blindly. Thus providing an improvement compared to the previous article of this same project of Miranda et al. (2019).

This opens a horizon for new challenges where, if the size of the data set and the computational capabilities of the system are not optimal, as is often the case in specific systems such as the trap described, combined ML techniques can be explored for image classification on remote devices.

In addition to the benefits described above, the application of ML strategies opens up new possibilities for the system. Once the model is trained, the device performs the prepreocessing and inference on the image data, but only the prediction is exchanged with the server. In this regard, it is also worth mentioning the advantages in terms of privacy, e.g. there is no risk related to identifying people in images sent to the server. Since no images are shared with the server, it also represents an improvement in terms of privacy. Moreover, these models are relatively small compared to state-of-the-art neural networks and might be running on small IoT devices, such as Raspberry Pi B+ used in this case, or even smaller very low power microcontroller boards. Overall, it implies a reduction in power and energy consumption and an increase in battery life. All this is possible by making a more efficient use of bandwidth.

Finally, it is important to note that the data source used has come from a single e-trap system, so the system has the potential to increase the accuracy of the results as the system of nodes grows while each e-trap system can learn specific details of the conditions that make it unique.

## 8 Conclusions

The main contributions of this study are threefold: development of an intelligent system for efficient crop monitoring, demonstrating superior performance of ML methods over DL for this particular case study, and further improving performance using a simple model ensembling approach.

An intelligent system capable of detecting the olive fly using non-invasive techniques was successfully developed. The system is capable of monitoring the fly and olive fly population using image processing and ML techniques. This enabled experts to remotely monitor the status and evolution of the fly population, thereby reducing the need for manual fly counts in the fields.

Since a relatively small dataset was available, the application of classical ML techniques worked better compared to a transfer learning approach using pre-trained DL models. The study revealed that classical ML models (RF and SVM) outperformed CNN solutions in this case. Despite the scarcity of images, these models demonstrated good accuracy, making them an attractive option for resource-constrained applications. In particular, the RF and SVM models reported an accuracy of 62.1% and 86.4% for the olive fly detection task, respectively. In addition, the RF and SVM approaches reported an accuracy of 91.9% and 94.5%, respectively, when classifying only flies, regardless of the species.

Finally, the model performance was further improved by combining both RF and SVM models. RF was found to be more sensitive in detecting a potential fly infestation, while SVM demonstrated a more cautious approach in stating whether a fly was present in the sticky trap. As a result, combining both models led to an increased accuracy of 89.1% for the olive fly detection task.

In conclusion, this research showcases the successful implementation of ML in an e-trap system for olive fly detection, providing valuable insights and benefits. The combination of RF and SVM models demonstrated promising results, offering more efficient crop monitoring and control tasks to the experts. The potential for using small IoT devices for image classification opens up new possibilities, emphasizing the significance of ML in optimizing resource usage and enhancing privacy protection. As the system grows by increasing the number of e-traps, more data will be available. Therefore, it holds the potential to further enhance accuracy by learning from multiple e-trap systems, making it a promising tool for effective and sustainable fly population management.

## Data availability statement

The raw data supporting the conclusions of this article will be made available by the authors, without undue reservation.

## Author contributions

MM-R, MM and BA-L contributed to conception of the study. MM-R and BA-L contributed to the design of the study MM-R organized the datasets and performed the experimental analysis. MM-R wrote the first draft of the manuscript. MM-R, MM, AM, and BA-L wrote sections of the manuscript. All authors contributed to manuscript revision, read, and approved the submitted version.
